# Macrophage type modulates osteogenic differentiation of adipose tissue MSCs

**DOI:** 10.1007/s00441-017-2598-8

**Published:** 2017-03-30

**Authors:** Yang Zhang, Thomas Böse, Ronald E. Unger, John A. Jansen, Charles James Kirkpatrick, Jeroen J. J. P. van den Beucken

**Affiliations:** 1 Department of Biomaterials (309), Radboudumc, PO Box 9101, 6500HB Nijmegen, The Netherlands; 2 REPAIR-lab, Institute of Pathology, Johannes Gutenberg University Mainz, Mainz, Germany

**Keywords:** Macrophage, MSC, Osteogenic differentiation, Cell culture model

## Abstract

Since the reconstruction of large bone defects remains a challenge, knowledge about the biology of bone healing is desirable to develop novel strategies for improving the treatment of bone defects. In osteoimmunology, macrophages are the central component in the early stage of physiological response after bone injury and bone remodeling in the late stage. During this process, a switch of macrophage phenotype from pro-inflammatory (M1) to anti-inflammatory (M2) is observed. An appealing option for bone regeneration would be to exploit this regulatory role for the benefit of osteogenic differentiation of osteoprogenitor cells (e.g., mesenchymal stem cells; MSCs) and to eventually utilize this knowledge to improve the therapeutic outcome of bone regenerative treatment. In view of this, we focused on the in vitro interaction of different macrophage subtypes with adipose tissue MSCs to monitor the behavior (i.e. proliferation, differentiation and mineralization) of the latter in dedicated co-culture models. Our data show that co-culture of MSCs with M2 macrophages, but not with M1 macrophages or M0 macrophages, results in significantly increased MSC mineralization caused by soluble factors. Specifically, M2 macrophages promoted the proliferation and osteogenic differentiation of MSCs, while M0 and M1 macrophages solely stimulated the osteogenic differentiation of MSCs in the early and middle stages during co-culture. Secretion of the soluble factors oncostatin M (OSM) and bone morphogenetic protein 2 (BMP-2) by macrophages showed correlation with MSC gene expression levels for OSM-receptor and BMP-2, suggesting the involvement of both signaling pathways in the osteogenic differentiation of MSCs.

**Electronic supplementary material:**

The online version of this article (doi:10.1007/s00441-017-2598-8) contains supplementary material, which is available to authorized users.

## Introduction

Bone defects resulting from trauma, cancer and fractures represent a significant clinical problem for over 9 million people worldwide each year (Johnell and Kanis 2006). The treatment of these bone defects relies predominantly on transplantation of autografts or allografts, and to a lesser extent on the use of synthetic biomaterial scaffolds. In order to improve the efficacy of synthetic biomaterial scaffolds, major efforts have focused on cell-based constructs that combine such synthetic biomaterial scaffolds with cells from the patients (Ma et al. 2014a, b).

Most cell-based constructs focus on the use of (adult) stem cells, generally isolated as so-called mesenchymal stromal cells (MSCs) from either bone marrow or adipose tissue. It is remarkable that, in view of the chronological order of wound and bone healing, signaling molecules and cells involved in the processes prior to wound healing are largely ignored. In the natural healing process of damaged tissue, three distinct but overlapping stages occur from a few hours to several weeks (Dimitriou et al. 2005): (1) the early inflammatory stage; (2) the repair stage; and (3) the late remodeling stage. It is reasonable to speculate that the inflammatory response, which is evoked by the host immune system, initiates and primes the later bone repair process. In fact, the immune and skeletal systems have been reported to share a number of signaling molecules and regulatory networks (Takayanagi 2007). Research on the topic of bone remodeling has shown the influence of the immune system on bone healing success (Schlundt et al. 2015a, b) and has led to the emergence of “osteoimmunology” (Takayanagi 2007), which identifies the immune system as a potential tool for new therapeutic approaches to bone healing. Among the cells of the innate immunity, macrophages are recognized as key elements for the orchestration of the processes to re-establish tissue integrity and function after damage (Cho et al. 2014; Lavine et al. 2014). Thus, for instance, inflammatory bone disorders generally resulted in increased bone resorption and decreased bone formation (Hardy and Cooper 2009), and ablation of macrophages has been shown to inhibit intramembranous bone healing (Alexander et al. 2011). Nevertheless, the fundamentals of macrophage involvement in the behavior of osteoprogenitor cells and bone formation remain unclear.

It is well documented that, following bone injury, monocytes are rapidly recruited to the injury site and differentiate into macrophages, where they persist throughout the bone repair process (Glynne Andrew et al. 1994; Wu et al. 2013). In supporting the multiple events occurring during the healing process, versatile subtypes of macrophages have been distinguished depending on the environmental stimuli (Gordon 2003). M0 macrophages, after in vitro differentiation from monocytes by macrophage–colony-stimulating factor (M-CSF) or phorbol-12-myristate-13-acetate (PMA), are mature macrophages with larger and more flattened morphology compared to monocytes (Zajac et al. 2013). Further, two macrophage phenotypes are present as extremes of a continuum of functional states. M1 macrophages, in vitro classically polarized by lipopolysaccharide (LPS) and interferon gamma (IFN-γ), present a pro-inflammatory profile with high antigen-presenting capacity and increased secretion of pro-inflammatory cytokines (e.g., interleukin 1β, IL-1β, and tumor necrosis factors alpha, TNF-α). In contrast, M2 macrophages, alternatively polarized by interleukin 4 (IL-4) and interleukin 13 (IL-13), secrete high levels of anti-inflammatory cytokines (e.g., transforming growth factor beta, TGF-β and interleukin 10, IL-10), regulate and scavenge debris, and promote angiogenesis and tissue remodeling (Gordon 2003). Recent studies depicted a switch in macrophage subtype from the pro-inflammatory M1 subtype to the anti-inflammatory M2 subtype during the bone healing process (Tasso et al. 2013; Wu et al. 2015), suggesting differential roles of these macrophage subtypes and their secreted cytokines on the recruitment, proliferation and differentiation of MSCs. However, more detailed information is required to clarify macrophage contribution to the osteogenic differentiation of MSCs.

The objective of this study was to elucidate the effect of different macrophage subtypes on the osteogenic differentiation of MSCs in co-culture models. We hypothesized that M2 macrophages are able to promote the osteogenic differentiation of MSCs, while M0 and M1 macrophages inhibit this process. Therefore, human monocytes were activated and/or polarized into M0, M1 and M2 macrophages and then directly co-cultured with human MSCs at different ratios to determine their osteogenic capacities. Further studies on the mechanism by which these macrophages affect MSCs were performed by an indirect co-culture set-up to reveal paracrine effects of macrophage subtypes on the behavior of MSCs and to identify the involved signaling molecules.

## Materials and methods

### Reagents and cells

Dulbecco’s Modified Eagle Medium (α-MEM), RPMI-1640 medium, 1% penicillin-streptomycin (1% P/S) was purchased from Gibco (GrandIsland, USA). Fetal bovine serum (FBS), bovine serum albumin (BSA), trypsin, basic fibroblast growth factor (bFGF), PMA, LPS, IFN-γ, IL-4, IL-13, glycerol 2-phosphate disodium salt hydrate (β-glycerophosphate), dexamethasone, and ascorbic acid were purchased from Sigma-Aldrich (St. Louis, USA). Collagenase was purchased from Roche Diagnostics (Mannheim, Germany). TNF-α and TGF-β ELISA kits were purchased from eBioscience (San Diego, USA). BMP-2 and OSM ELISA kits were purchased from R&D systems (San Diego, USA). Monoclonal anti-human CCR7 antibody was purchased from Abcam (Cambridge, UK), mouse purified anti-human CD36 was from Biolegend (San Diego, USA) and mouse anti-human CD68 was from Dako (Heverlee, Belgium). All secondary antibodies and 4, 6-diamidino-2-phenylindole (DAPI) was purchased from Invitrogen (Waltham, USA). All cell culture flasks and plates were purchased from Greiner Bio-one (Frickenhausen, Germany).

Human adipose tissue MSCs were isolated and expanded as previously described (Ma et al. 2013). In brief, human subcutaneous adipose tissue was obtained from the Department of Plastic Surgery (Radboudumc, Nijmegen, the Netherlands) after informed consent. Obtained adipose tissue was minced using surgical scalpels and washed with PBS. The aspirated lipid fraction was diluted with an equal volume of 0.1% collagenase digestion solution and then incubated at 37 °C for 60 min under rotation (250 rpm). After Ficoll density centrifugation (600 g for 10 min), the cell pellet was resuspended and filtered through a 100- μm cell strainer. Mononuclear cells were adjusted to 1 × 10^7^ cells per 15 ml and then cultured in 10% FBS, 1% PS, and 1 ng/ml of bFGF supplemented α-MEM. The attached cells were designated as adipose tissue MSCs and characterized by positive expression of CD73, CD90 and CD105 and negative expression of CD45 (Hayrapetyan et al. 2016). Cells in passage 3–5 from three donors were used in this study.

The human monocytic cell line (THP-1) was purchased from the American Type Culture Collection (Manassas, USA) and cultured in RPMI-1640 medium supplemented with 10% heat-inactivated FBS and 1% P/S.

### Polarization and characterization of macrophages

THP-1 cells were differentiated and polarized according to established protocols (Freytes et al. 2013; Stewart et al. 2012). Briefly, 1 × 10^6^ cells were plated in 6-well plates with 3 ml culture medium plus 25 ng/ml PMA for 48 h to activate monocytes into M0 macrophages. For polarization, M0 macrophages were treated for another 48 h, either with an addition of 20 ng/ml IFN-γ and 100 ng/ml LPS to obtain M1 macrophages or with 20 ng/ml IL-4 and 20 ng/ml IL-13 to obtain M2 macrophages. Conditioned medium from polarized macrophages was used for measuring TNF-α, TGF-β and IL-10 via ELISAs following the instructions of the manufacturer. Activated and polarized macrophages were fixed with 4% paraformaldehyde and then subjected to immunocytochemistry. The M1 macrophage marker CCR7 (Stewart et al. 2012) and M2 macrophage marker CD36 (Stewart et al. 2012) were stained with the primary antibodies, rabbit monoclonal anti-human CCR7 and mouse purified anti-human CD36, respectively, for 2 h in PBS with 1% BSA. Cells were then washed and incubated for 1 h with goat anti-mouse Alexa-488 labeled IgG and donkey anti-rabbit Alexa-568 labeled IgG in the dark. After washing, cells were stained with DAPI for 5 min. Immunofluorescence images were acquired with a fluorescence microscope (Zeiss AxioCam MRc5; Carl Zeiss Microimaging, Germany) and the relative intensity of fluorescence was analyzed using ImageJ (U.S. National Institutes of Health, Bethesda, USA). The values of red (Alexa-568) and green (Alexa-488) fluorescence of each sample were further normalized to the value of blue fluorescence (DAPI).

### Direct co-culture of macrophages and MSCs

#### Direct co-culture of macrophages and MSCs at different ratios

THP-1 cells were differentiated and polarized into the various subtypes of macrophages (i.e. M0, M1, and M2) as described above. Macrophages were detached by trypsin and counted by a hemocytometer (LO-Laboroptik, Friedrichsdorf, Germany). Then, 8 × 10^4^, 2 × 10^4^ and 5 × 10^3^ M0, M1 or M2 macrophages were plated into 24-well plates, in which 2 × 10^4^ adipose tissue MSCs had been seeded 6 h before. A mixture of THP-1 cell culture medium and osteogenic medium (mixture medium, 10% heat-inactivated FBS with 10 nM dexamethasone, 100 μM ascorbic acid, and 10 mM β-glycerophosphate) was used and refreshed every 3 days.

#### Immunostaining of direct co-culture

Cells were seeded on plastic coverslips (13 mm; Thermanox, MA, USA) in 24-well plates. After 4 weeks, the coverslips with attached cells (8 × 10^4^ macrophages groups) were washed with PBS and then fixed with 4% paraformaldehyde, followed by blocking with 1% BSA. Cells were then stained with mouse anti-human CD68 and goat anti-mouse Alexa-488 labeled IgG and DAPI. After staining and mounting, coverslips were imaged with a fluorescence microscope (Keyence International, Mechelen, Belgium). The number of macrophages and MSCs were counted based on nuclear staining (shape; macrophage nuclei were round; MSC nuclei were elongated) and CD68-positive cells (macrophages) by counting four random fields per well (magnification ×400). Cell densities were then normalized to the area of the fields.

#### Mineralization of direct co-culture

For mineralization tests, direct co-cultures were maintained for 4 weeks, washed twice with PBS, and incubated overnight with 1 ml 0.5 N acetic acid on a shaking table at room temperature. The calcium content of each well was quantified by a calcium assay as described previously (Ma et al. 2013).

### Indirect co-culture of macrophages and MSCs

#### Indirect co-culture of macrophages and MSCs by a transwell system

A total of 8 × 10^4^ macrophages were plated into 0.4-μm pore inserts of 24-well transwell plates in 200 μl of mixture medium (1:1 THP-1 cell culture medium and osteogenic medium), with 800 μl mixture medium containing 2 × 10^4^ MSCs added to the bottom of the well. Medium was changed on day 3, day 7 and then twice a week. After 2 and 4 weeks, MSCs were stained with Alizarin Red or quantified by calcium tests. In parallel samples, MSCs were collected for DNA content and ALP activity test and stained with ALP dyes after 7, 14, and 28 days. Additionally, medium from each group was collected at days 3, 7, 14, and 28 for protein analysis and MSCs were homogenized with 350 μl lysis buffer and then stored at −80 °C for RT-PCR.

#### DNA content of MSCs

Cell proliferation for MSCs was assessed using the PicoGreen DNA quantification assay kit, (Invitrogen). Cell layers were washed twice with PBS, after which 1 ml MilliQ water was added. Following two freeze–thaw cycles, samples were used for DNA quantification according to the instructions of the manufacturer.

#### ALP activity of MSCs

The ALP activity was measured using the same samples as used for cellular DNA content. A *p*-nitrophenyl phosphate (4-NP) method was adapted as developed previously (Ma et al. 2013). ALP activity results were normalized for DNA (expressed as nmol 4-NP/ng DNA/h). In addition, 2 parallel samples from each group were fixed with 4% paraformaldehyde and then histochemically stained in methanol using the Leukocyte Alkaline Phosphatase Kit (Sigma-Aldrich, St. Louis, USA) per the manufacturer’s protocol.

#### Mineralization of MSCs

The calcium content for indirect co-cultures was quantified with the same method as described for direct co-cultures. In parallel, 2 samples from each group were fixed in 4% paraformaldehyde at indicated time points and then stained with 1 ml/well alizarin red solution for 15 min at room temperature using an osteogenesis quantification kit (EMD Millipore, Billerica, USA). Stained samples were then photographed with a microscope (Keyence International).

#### Osteogenic gene expression of MSCs

mRNA of cells was extracted using the RNeasy Mini Kit (Qiagen, Valencia, USA) per the manufacturer’s protocol. After isolation, RNA was quantified using a Nanodrop ND1000 Spectrophotometer (Thermo Scientific, Hudson, USA). cDNA was generated from 1 μg of RNA using the SuperScript III reverse transcription kit (Invitrogen). For the RT-PCR reaction, 2 μl cDNA, 12.5 μl Mastermix (Life Technologies, Waltham, USA) and 3 μl primer mix with specific forward and reverse primers (Table S1) and 7.5 μl RNAse-free water was mixed. PCR reactions were performed and monitored using an ABI Prism 7700 Sequence Detection System (Perkin-Elmer/Applied Biosystems, Rotkreuz, Switzerland). The level of gene expression was calculated via the ∆∆Ct method (Schmittgen and Livak 2008). Four independent samples were used for each gene of interest.

### Osteogenic factors involved in macrophage and MSC interaction

#### Protein quantification of osteogenic factors

Medium collected from indirect co-culture at indicated time points and conditioned medium from polarized macrophages were used for BMP-2 and OSM ELISAs following the manufacturer’s instructions. Colorimetric changes were measured using a multi-mode spectrophotometer (Biotek, Winooski, USA).

#### Gene expression of osteogenic factors in MSCs and polarized macrophages

MSCs and different types of macrophages were homogenized with lysis buffer at indicated time points and mRNA was isolated as described above. RT-PCR was conducted with the same protocol, and gene expression of BMP-2, OSM and OSMR were quantified using primers listed in Tab. S1.

### Statistical analysis

Data are expressed as the mean and standard deviation (±SD). Statistical analysis was performed by GraphPad Prism v.5 (GraphPad Software, San Diego, USA) using one-way analysis of variance (ANOVA) with Dunnett’s post-test where multiple results were compared against a control, or with Bonferroni’s test for multiple comparisons. Two-way ANOVA with Bonferroni’s test was performed where two independent variables were present. Probability values of *P* < 0.05 were considered statistically significant.

## Results

### Characterization of polarized macrophages

**Fig. 1:**
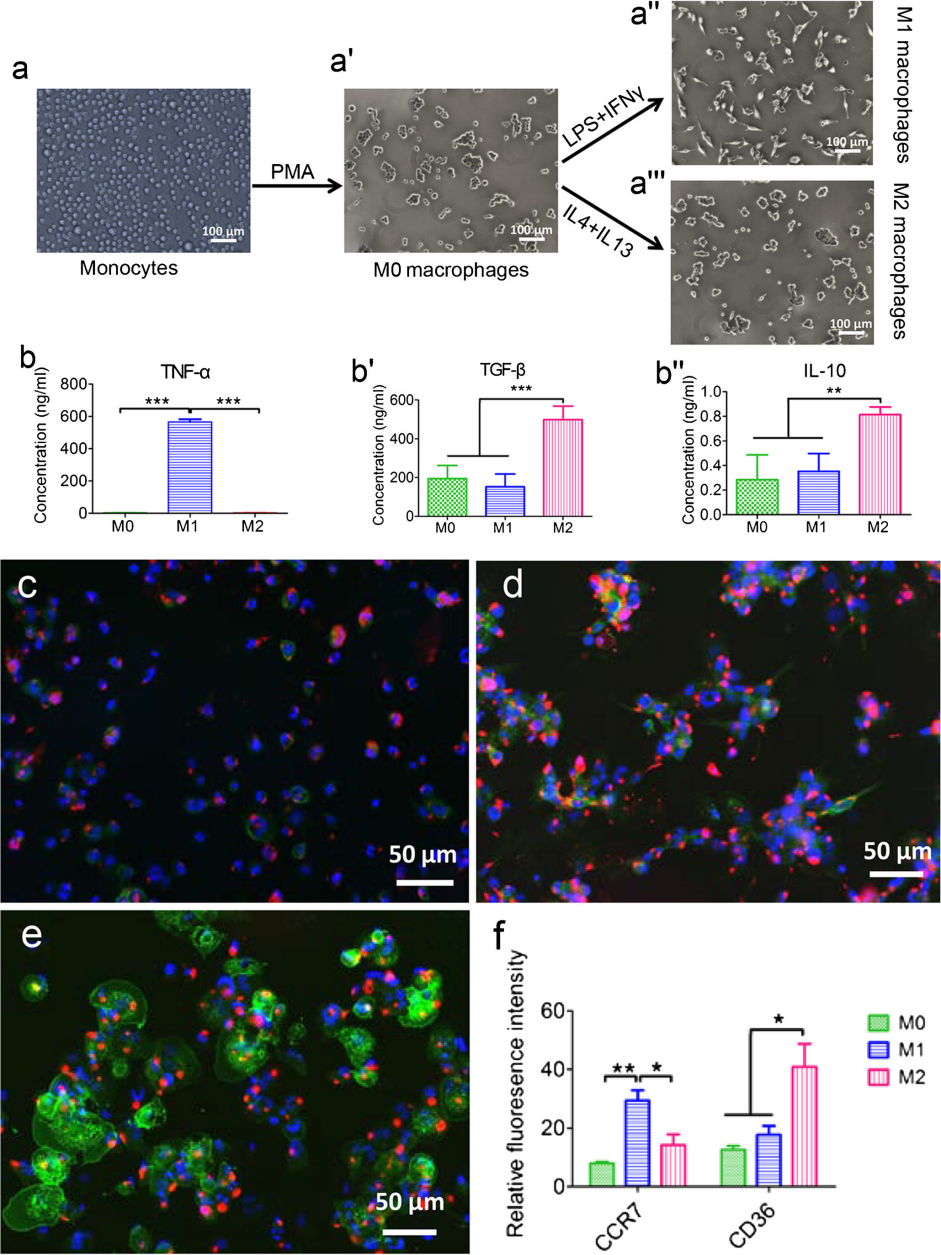
The polarization method and cytokine expression of M0, M1 and M2 macrophages derived from THP-1 monocytes. The schematic figure shows the differentiation of monocytes into polarized macrophages and their morphological appearance (**a**–** a'''**). TNF-α (**b**), TGF-β (**b'**) and IL-10 (**b''**) production in M0, M1 and M2 macrophages conditioned medium were assessed by ELISA. M0 (**c**), M1 (**d**) and M2 (**e**) macrophages were stained with M1-marker CCR7 (*red*), M2-marker CD36 (*green*) and DAPI (*blue*), respectively. The relative fluorescence intensity of CCR7 and CD36 were quantified by ImageJ (**f**). Statistical analysis was performed by one-way ANOVA with Bonferroni’s test. *P ≤ 0.05; ** *P* ≤ 0.01, *** *P* ≤ 0.001

THP-1 monocytes were activated with PMA to generate M0 macrophages, which made the cells adherent to plastic. M0 macrophages were further polarized into M1 and M2 macrophages in the presence of the appropriate cytokines (Fig. 1a). Cytokine secretion profiles for TNF-α, TGF-β, and IL-10 depended on macrophage subtype (Fig. 1b–b''). Significantly higher TNF-α secretion was observed for M1 macrophages (565.74 ± 17.58 ng/ml) compared to M0 macrophages (3.52 ± 1.55 ng/ml; *P* < 0.001) and M2 macrophages (3.06 ± 0.74 ng/ml; *P* < 0.001). In contrast, significantly higher TGF-β secretion was observed for M2 macrophages (499.32 ± 69.48 ng/ml) compared to M0 macrophages (193.56 ± 68.74 ng/ml; *P* < 0.001) and M1 macrophages (151.69 ± 66.49 ng/ml; *P* < 0.001). Similarly, IL-10 secretion was significantly higher for M2 macrophages (0.82 ± 0.06 ng/ml) compared to both M0 macrophages (0.28 ± 0.20 ng/ml; *P* < 0.01) and M1 macrophages (0.35 ± 0.14 ng/ml; *P* < 0.01).

Immunostaining for macrophage subtype markers showed mixed populations of M1 and M2 macrophages after polarization procedures (Fig. 1c–e). M1 polarization showed macrophages with positive staining for the M1 marker, CCR7, and slightly less positive staining for the M2 marker, CD36. In contrast, M2 macrophages showed enhanced positive staining for CD36 and less positive staining for CCR7. Quantification of the fluorescent signal from macrophage polarization marker immunoreactions showed obvious differences between the three macrophage phenotypes (Fig. 1f). CCR7 was significantly higher expressed in M1 macrophages (29.44 ± 3.55; *P* < 0.05), compared to M0 (7.96 ± 0.57) and M2 macrophages (14.27 ± 3.61). In contrast, CD36 was significantly higher expressed in M2 macrophages (40.94 ± 5.81) compared to M0 (12.70 ± 1.22) and M1 macrophages (17.72 ± 3.05).

### Direct co-culture of macrophages and MSCs

Experiments were performed with MSCs isolated from three different donors. As similar results were obtained from these experiments, the results below mainly describe the data from donor 3 as the representative experiment.

#### Cell distribution

**Fig. 2:**
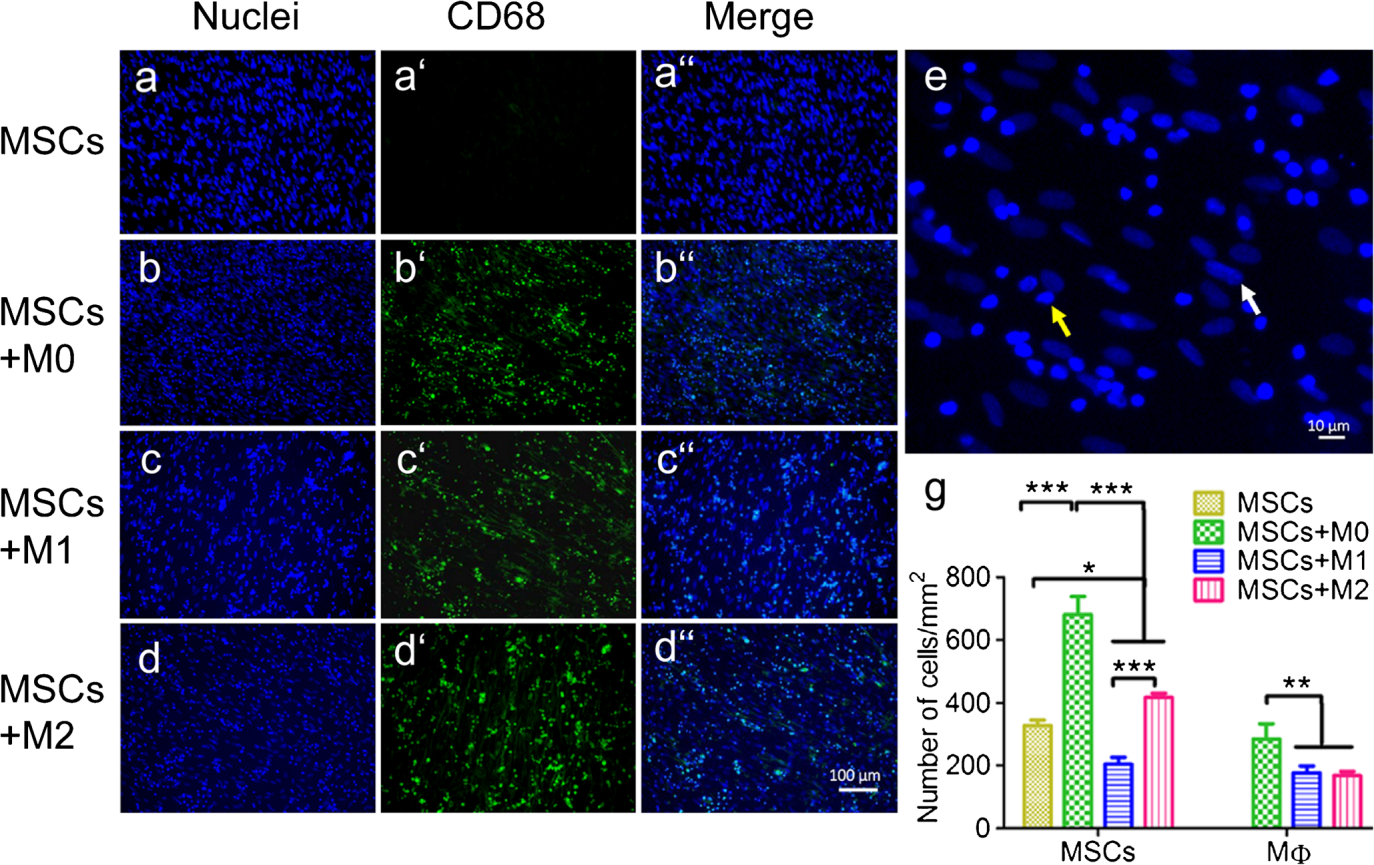
Immunostaining of different types of macrophages (MФ) co-cultured with MSCs. MSCs were monocultured or co-cultured with M0, M1 and M2 macrophages (1:4 ratio) for 4 weeks and stained with DAPI (*blue*, **a**–**d**), pan-macrophage marker CD68 (*green*, **a'**–**d'**), and then merged (**a''**–**d''**). The number of macrophages and MSCs was counted based on shape of nuclei (**e**) and quantified (**g**). *White arrow* indicates the MSC while *yellow arrow* indicates the macrophage. Statistical analysis was performed by one-way ANOVA with Bonferroni’s test. *n* = 4, *P ≤ 0.05, ** *P* ≤ 0.01, *** *P* ≤ 0.001

After 4 weeks of direct co-culture, pan-macrophage staining (CD68) combined with nuclear staining (DAPI) showed a homogeneous distribution of both cell types (Fig. 2a–a'', b–b'', c–c'', d–d''). Although an equal number of macrophages were initially seeded for the co-cultures, higher numbers of M0 macrophages (592 ± 101/mm^2^; *P* < 0.05) were observed during the co-culture compared to both M1 (368 ± 45/mm^2^) and M2 (351 ± 27/mm^2^) macrophages with MSCs (Fig. 2e, g). Simultaneously, MSCs co-cultured with M0 (681 ± 57/mm^2^) and M2 (418 ± 13/mm^2^) macrophages showed significantly higher numbers compared to the MSCs monoculture (327 ± 17/mm^2^; *P* < 0.05), while MSCs number sdecreased with M1 macrophages (204 ± 22/mm^2^; *P* < 0.05).

#### Mineralization

**Fig. 3:**
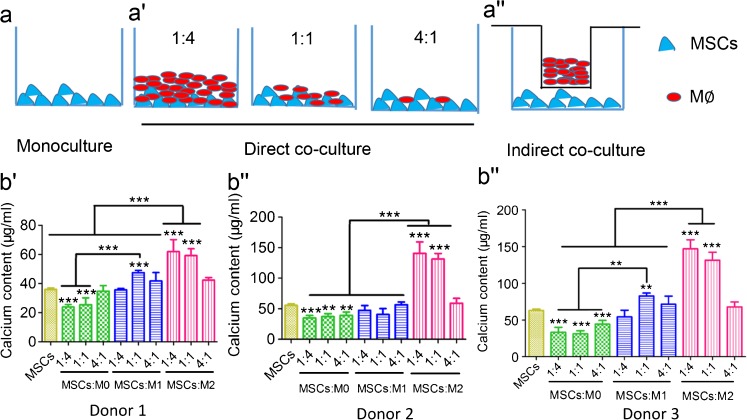
Mineralization of MSCs directly co-cultured with M0, M1 and M2 macrophages. The schematic diagram shows the set-up of MSCs monoculture (**a**), direct co-culture (at different ratios; **a'**) or indirect co-culture (at ratio of 1:4; **a''**) with M0, M1 and M2 macrophages respectively. Mineralization capacity of monoculture and direct co-culture from 3 donors was assessed by calcium content after 4 weeks in osteogenic medium (**b**–**b''**). Statistical significance relative to controls (MSCs monoculture) and between groups was determined by two-way ANOVA with Bonferroni’s test correction, *n* = 5, **P ≤ 0.01, *** *P* ≤ 0.001. *Asterisks* on the *top of the columns* indicate significant differences from the MSCs control

Calcium content measurements were used to determine the mineralization capacity of MSCs co-cultured with macrophages and MSCs monoculture controls. Different ratios of MSCs to macrophages and two co-culture methods, namely direct co-culture and indirect co-culture by using the transwell system, were used here to investigate this effect (Fig. 3a–a''). The calcium content from direct co-cultures showed macrophage subtype-dependent promoting effects on MSCs, irrespective of donors (Fig. –b''). Taking 3 donors, for example (Fig. 3b''), M0 macrophages decreased the mineralization of co-cultured MSCs to around 40 μg/ml, irrespective of the macrophage to MSC ratio. M1 macrophages, however, showed different effects on the mineralization of MSCs depending on their ratio. M1 macrophages and MSCs at a ratio of 1:1 also enhanced the mineralization (72.75 ± 4.06 μg/ml) compared to MSCs controls (63.26 ± 1.77 μg/ml; *P* < 0.01), while this effect was not obvious for the ratios 1:4 and 4:1. In contrast, M2 macrophages significantly increased the mineralization of co-cultured MSCs, and this effect was propor-tional to the ratio of macrophages to MSCs. M2 macrophages to MSCs at 4:1 and 1:1 ratios reached significantly higher mineralization of 146.84 ± 12.31 μg/ml and 131.38 ± 10.94 μg/ml, respectively, compared to MSCs monoculture (63.26 ± 1.77 μg/ml; *P* < 0.001). At a ratio of 1:4, M2 macrophages reached a similar mineralization level (68.01 ± 6.59 μg/ml) compared to MSC monoculture (*p* > 0.05).

### Indirect co-culture of macrophages and MSCs

Experiments were performed with MSCs isolated from 3 different donors. As similar results were obtained from these experiments, the data below describe the data of donor 3 as representative experiment. More data from donor 1 and donor 2 are shown in Supplementary Fig. S1a, c and Fig. S1b, d.

#### Cell proliferation of MSCs

**Fig. 4:**
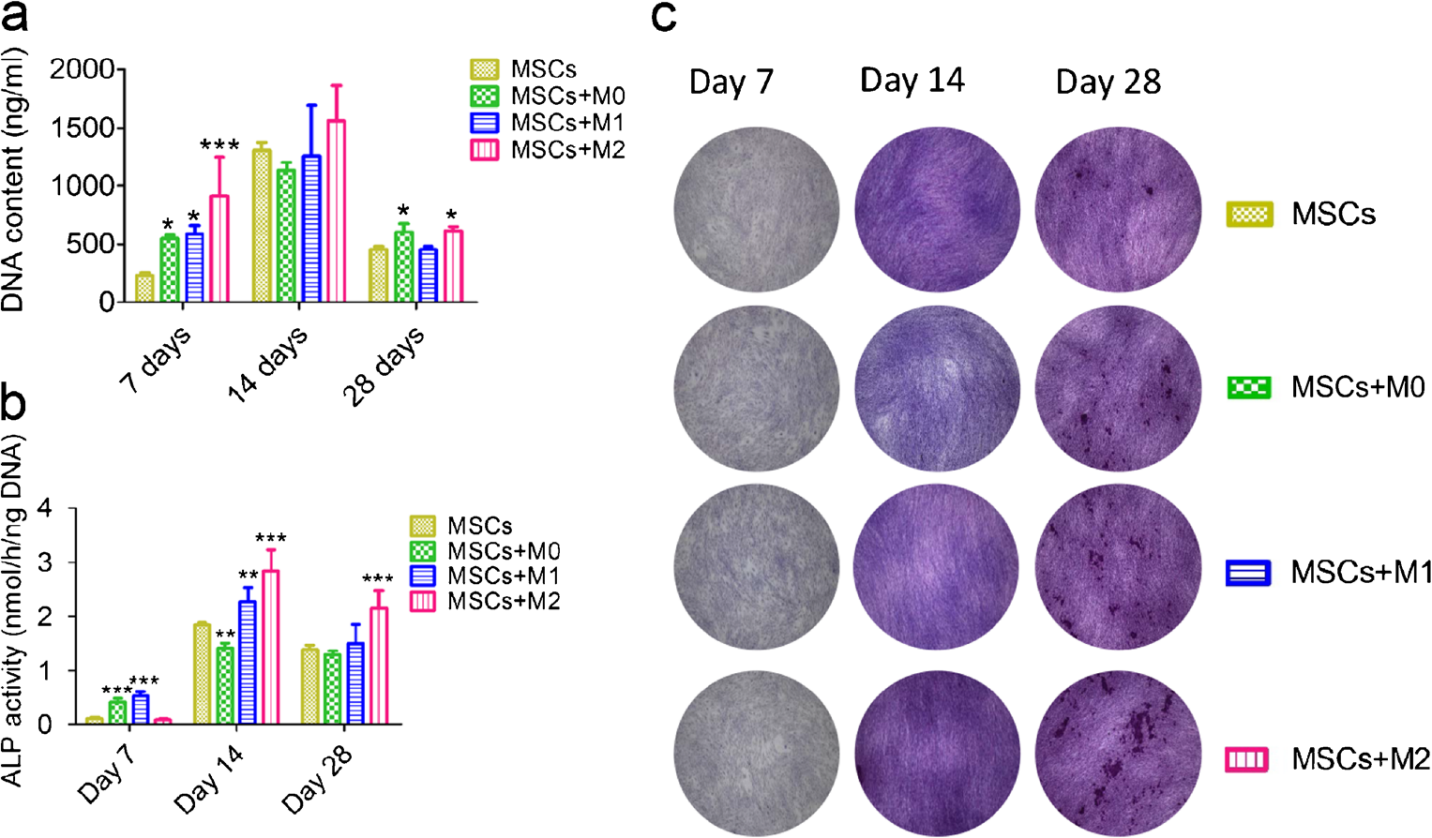
Cell proliferation and ALP activity of MSCs indirectly co-cultured with M0, M1 and M2 macrophages. MSCs were monocultured and indirectly co-cultured with three types of macrophages and their proliferation was determined by DNA content assay (**a**) and their osteogenic differentiation was determined by ALP-activity assay (**b**) and ALP staining (**c**). Statistical analysis was performed by one-way ANOVA with Dunnett’s post-test. *n* = 4, * *P* ≤ 0.05, ** *P* ≤ 0.01, *** *P* ≤ 0.001

DNA content of co-cultured MSCs was assessed to study effects of different types of macrophages on the growth of MSCs during co-culture. As shown in Fig. 4a, after 7 days, all the three types of macrophages increased the cell number of MSCs, with the effect of M2 macrophages on MSC proliferation being the highest (913.93 ± 334.15 ng/ml; *P* < 0.001), followed by MSCs with M1 macrophages (585.49 ± 74.20 ng/ml; *P* < 0.05) and M0 macrophages (547.22 ± 27.90 ng/ml; *P* < 0.05) compared to MSC monoculture (229.44 ± 21.42 ng/ml). After 14 days, although DNA content was increased in all groups, no difference was observed between MSC monoculture and co-cultures with macrophages. However, on day 28, M2 macrophages still significantly stimulated the growth of co-cultured MSCs (614.27 ± 36.53 ng/ml) compared to MSC monoculture (454.29 ± 26.63 ng/ml; *P* < 0.05) and MSCs with M1 macrophages (453.85 ± 27.04 ng/ml; *P* < 0.05).

#### ALP activity of MSCs

The activity of alkaline phosphatase in co-cultured MSCs was assessed as a marker of osteogenic differentiation. Generally, the level of endogenous ALP activity increased and peaked around 14 days and then decreased for all experimental groups (Fig. 4b, c). In view of different macrophage subtype effects, the co-culture of MSCs with M0 and M1 macrophages increased alkaline phosphatase activity to 0.42 ± 0.07 nmol/ng DNA/h and 0.54 ± 0.08 nmol/ng DNA/h, respectively, measured at day 7, compared to MSC monoculture (0.11 ± 0.02 nmol/ng DNA/h; *P* < 0.001) and with M2 macrophages (0.09 ± 0.01 nmol/ng DNA/h; *P* < 0.001) (Fig. 4b). However, after 14 days, M2 (2.85 ± 0.38 nmol/ng DNA/h; *P* < 0.001) and M1 macrophages (2.28 ± 0.30 nmol/ng DNA/h; *P* < 0.05) significantly increased the ALP activity in co-cultured MSCs. In contrast, MSCs co-cultured with M0 macrophages showed significantly less ALP activity (1.43 ± 0.09 nmol/ng DNA/h; *P* < 0.01) compared to MSCs monoculture. After 28 days, MSCs co-cultured with M0 and M1 macrophages showed similar ALP activity compared to MSC monoculture. In contrast, MSCs co-cultured with M2 macrophages (2.16 ± 0.32 nmol/ng DNA/h) still had a significantly higher ALP activity compared to all other experimental groups (*P* < 0.001; Fig. 4b, c).

#### Mineralization of MSCs

**Fig. 5:**
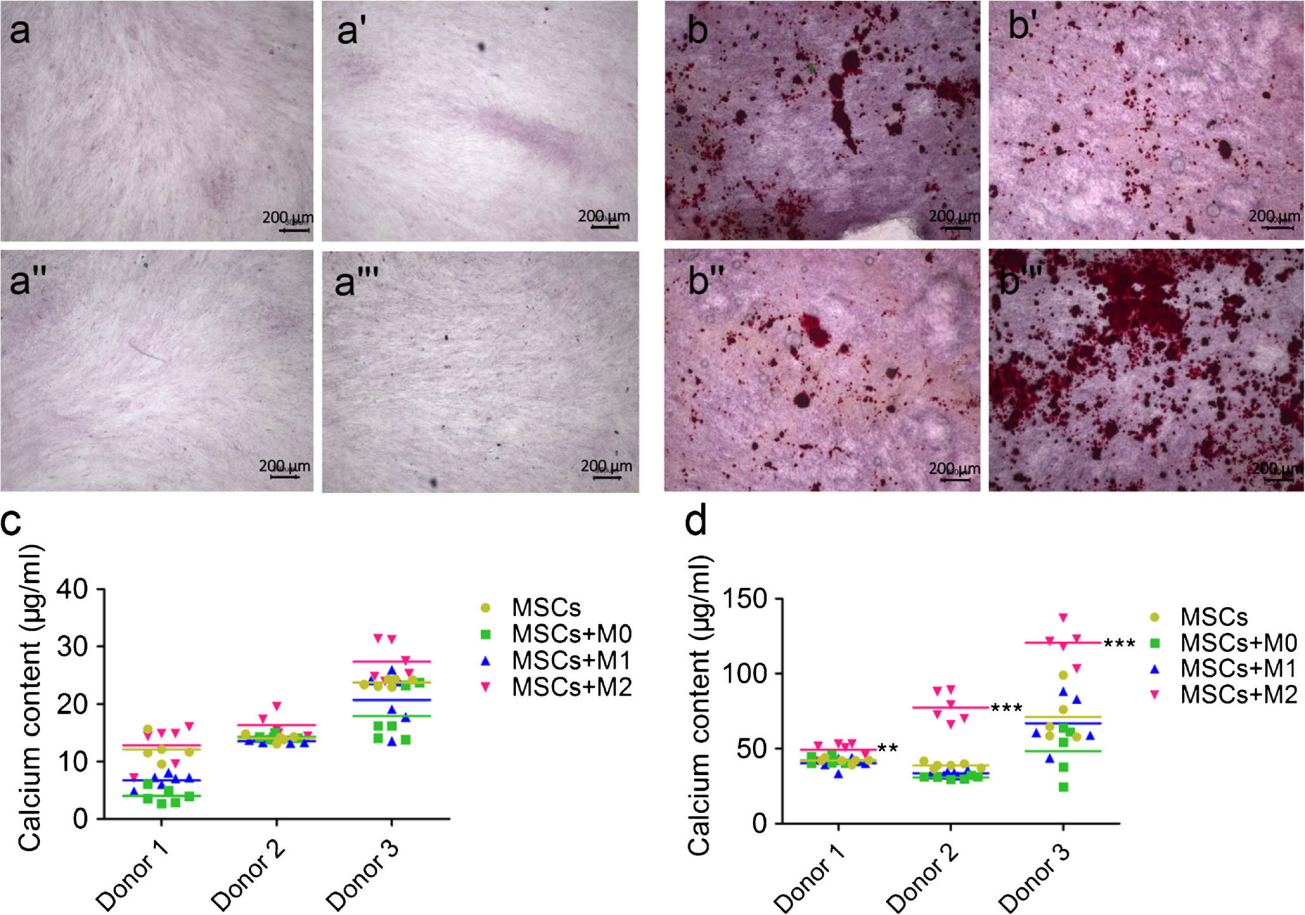
Mineralization of MSCs indirectly co-cultured with M0, M1 and M2 macrophages. MSCs monocultured (**a**,** b**) or indirectly co-cultured with M0 (**a'**,** b'**), M1 (**a'**, **b''**) and M2 (**a'''**,** b'''**) macrophages at a ratio of 1:4 were stained with alizarin red after 2 weeks (**a**–**a'''**) and 4 weeks (**b**–**b'''**), respectively. Calcium content of MSCs (3 different donors) monocultured or indirectly co-cultured with three types of macrophages in the osteogenic medium was determined after 2 weeks (**c**) and 4 weeks (**d**), respectively. Statistical analysis was performed by one-way ANOVA with Dunnett’s post-test. *n* = 5, *P ≤ 0.05, ** *P* ≤ 0.01, * **P ≤ 0.001

In indirect co-cultures using a transwell system (Fig. 3a''), MSCs co-cultured with M2 macrophages attained the apparent highest mineralization compared to other experimental groups, based on alizarin red staining. In contrast, the effect from M0 and M1 macrophages on the mineralization of MSCs was not obvious compared to MSC monoculture (Fig. 5a–a''', b–b'''). Quantitatively, 27.3 ± 3.26 μg/ml calcium was obtained after 2 weeks of indirect co-culture of M2 macrophages and MSCs (donor 3), which obtained similar levels (*p* > 0.05) as the other experimental groups (less than 20 μg/ml; Fig. 5c). After 4 weeks indirect co-culture, the calcium content of MSCs co-cultured with M2 macrophages reached 120.55 ± 10.09 μg/ml compared to MSC monoculture (71.30 ± 17.11 μg/ml; *P* < 0.001), MSCs with M0 (48.46 ± 16.70 μg/ml; *P* < 0.001) and M1 macrophages (67.03 ± 18.39 μg/ml; *P* < 0.001) (Fig. 5d).

#### Gene expression of osteogenic markers of MSCs in indirect co-culture

**Fig. 6:**
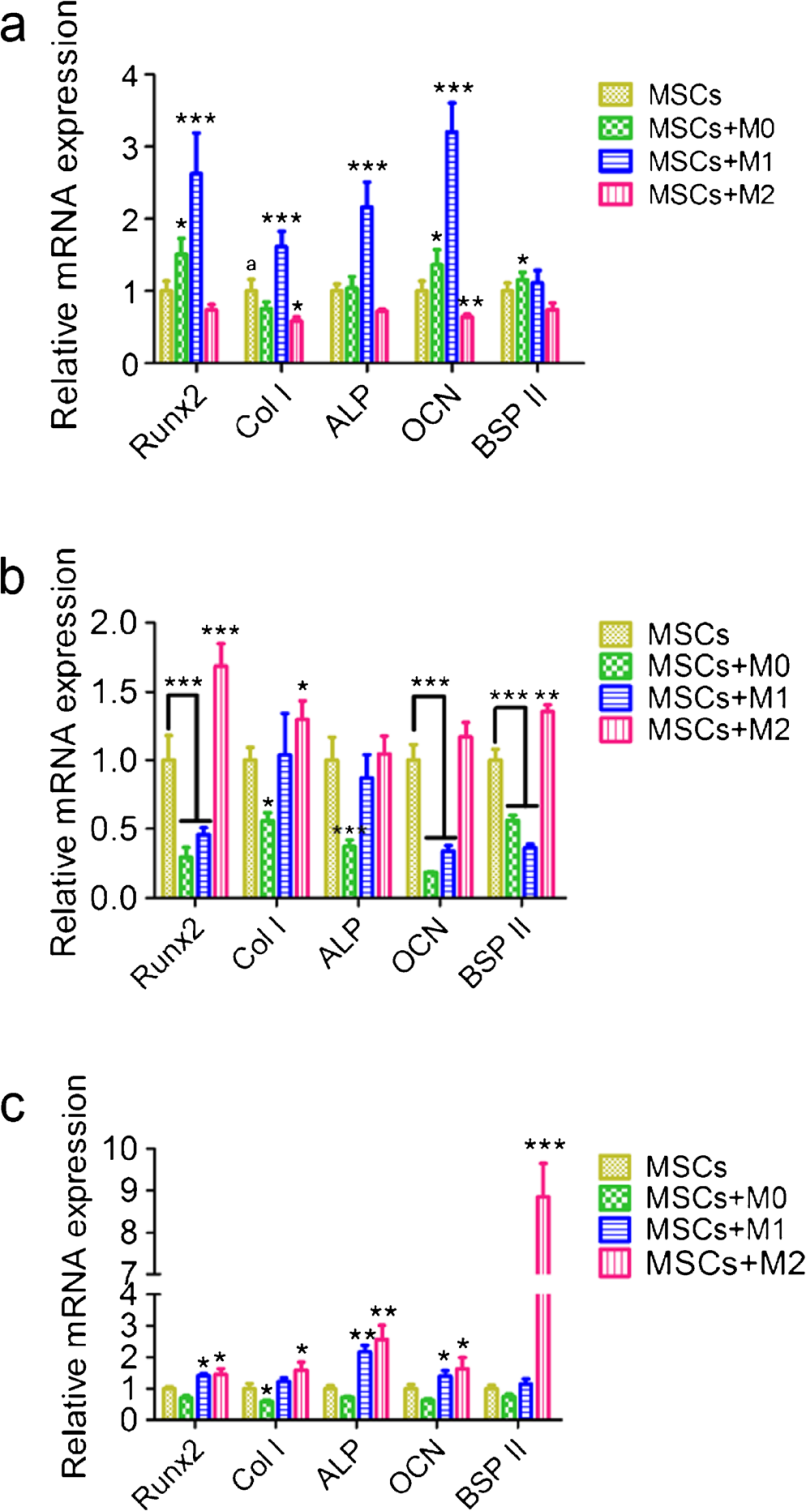
Gene expression of osteogenic markers by MSCs indirectly co-cultured with M0, M1 and M2 macrophages. MSCs were indirectly co-cultured with three types of macrophages and their key osteogenic gene expression was determined by RT-PCR after 3 days (**a**), 7 days (**b**) and 14 days (**c**). Statistical analysis was performed by one-way ANOVA with Dunnett’s post-test. *n* = 4, *P ≤ 0.05, ** *P* ≤ 0.01, *** *p* ≤ 0.001

Osteogenic differentiation of MSCs occurs along with an increase in the expression of osteogenesis-related genes, which were examined by RT-PCR analysis. After 3 days co-culture, the gene expression levels of runt-related transcription factor 2 (Runx2), collagen I, ALP, and osteocalcin (OCN) were 2∼3 times increased in MSCs indirectly co-cultured with M1 macrophages, in comparison to MSC monoculture (*P* < 0.001). M0 macrophages increased the expression of two osteogenic markers (Runx 2 and OCN), but to a lesser extent (*P* < 0.05). For co-cultures with M2 macrophages, this stimulating effect was not observed on day 3 (Fig. 6a).

On day 7, MSCs along with M0 and M1 macrophages inhibited the gene expression of Runx2, OCN and bone sialoprotein II (BSP II) (*P* < 0.001). By contrast, all osteogenesis-related genes, except for ALP, showed enhanced expression in MSCs co-cultured with M2 macrophages (Fig. 6b).

After 14 days, Runx2, ALP and OCN, were still more highly expressed in MSCs co-cultured with either M1 or M2 macrophages, compared to MSC monoculture (*P* < 0.05). Notably, BSP II expression was significantly enhanced (approximately 10-fold) for MSCs with M2 macrophages (*P* < 0.001), but not for MSCs co-cultured with M0 or M1 macrophages (*P* > 0.05) (Fig. 6c).

### Osteogenic factors involved in macrophage and MSC interaction

**Fig. 7:**
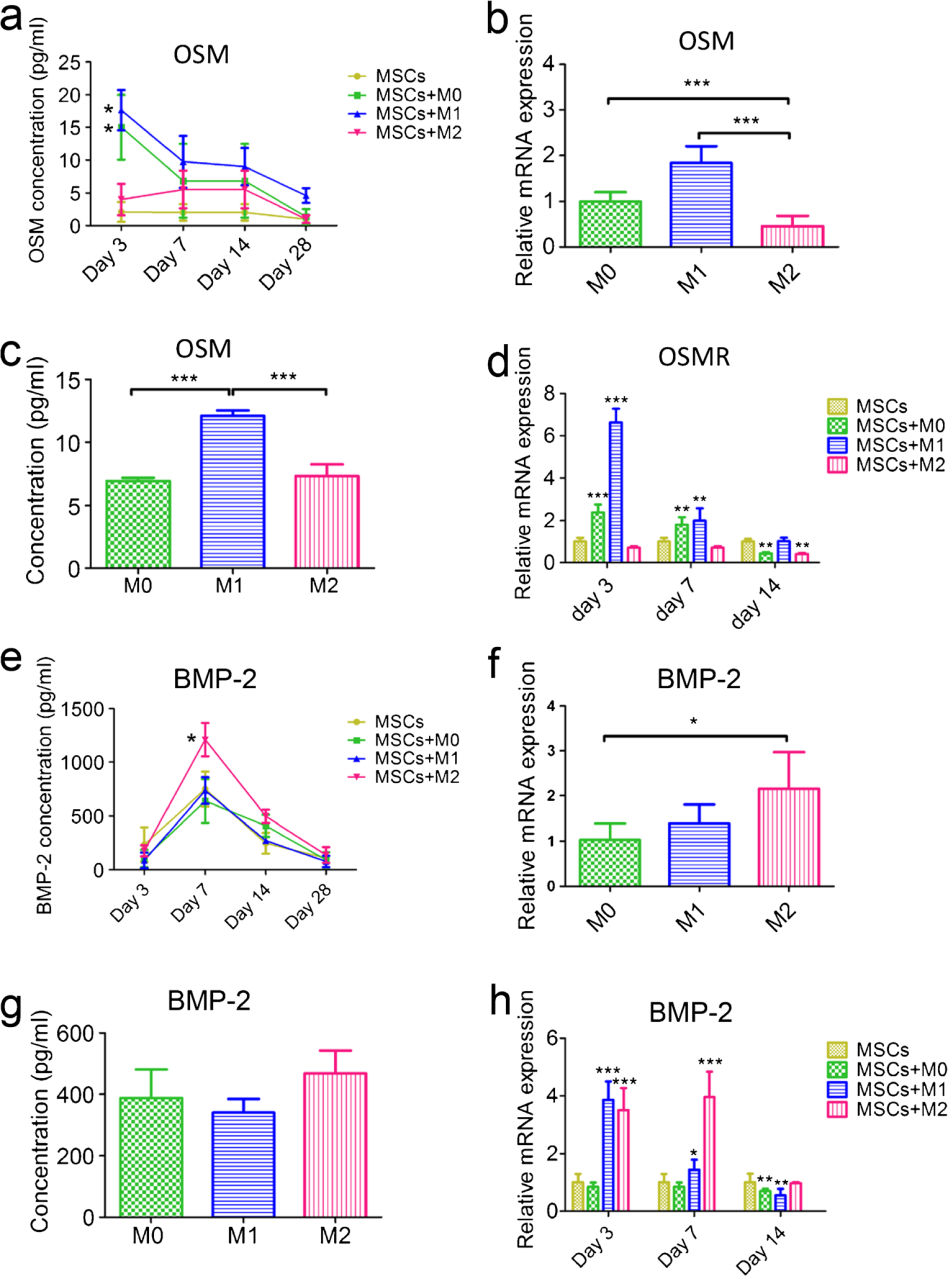
Secretion of key osteogenesis-related proteins in MSCs monoculture, MSCs and macrophages indirect co-cultures and macrophages conditioned medium. OSM secretion in macrophages and MSCs co-culture medium was assessed by ELISA (**a**). OSM gene expression of M0, M1 and M2 macrophages was determined by RT-PCR (**b**) and its protein secretion in conditioned medium was determined by ELISA (**c**). OSM receptor (OSMR) gene expression in MSCs was determined by RT-PCR (**d**). BMP-2 secretion in macrophages and MSCs co-culture medium was assessed by ELISA (**e**). BMP-2 gene expression of M0, M1 and M2 macrophages was determined by RT-PCR (**f**) and its protein secretion in conditioned medium was determined by ELISA (**g**). BMP-2 gene expression in MSCs was determined by RT-PCR (**h**). One-way ANOVA with Bonferroni’s correction was performed for comparison of OSM and BMP-2 gene expression and protein secretion between different types of macrophages. One-way ANOVA with Dunnett’s post-test correction was performed for comparison of OSM and BMP-2 concentration, OSMR and BMP-2 gene expression in MSCs. *n* = 4, *P ≤ 0.05, ** *P* ≤ 0.01, *** *P* ≤ 0.001

Two osteogenesis-related soluble proteins, OSM and BMP-2, were analyzed in the co-culture medium to evaluate potential involvement in the effects of macrophages on the osteogenic induction of MSCs (Fig. 7). Higher concentrations of OSM were found in the co-culture medium from M0-MSCs (15.02 ± 4.06 pg/ml) and M1-MSCs (17.63 ± 3.09 pg/ml) on day 3 compared to MSC monoculture (2.11 ± 1.52 pg/ml; *P* < 0.01) and M2-MSCs (4.01 ± 2.39 pg/ml; *P* < 0.01) (Fig. 7a). We further assessed OSM gene expression and protein secretion in three types of macrophages and their corresponding conditioned medium. M0 and M1 macrophages expressed almost twice to four times more OSM than M2 macrophages at the gene level (Fig. 7b). Secretion of OSM was also higher at the protein level in the conditioned medium of M1 macrophages (14.38 ± 3.42 pg/ml) compared to M2 macrophages (6.20 ± 3.06 pg/ml; *P* < 0.001) (Fig. 7c). The expression of the receptor of OSM (OSMR) was found to be enhanced in MSCs co-cultured with M0 (around 2-fold) and M1 macrophages (around 7-fold) after 3 days and 2-fold enhanced with M0 and M1 macrophages after 7 days (Fig. 7d) compared to MSC monoculture controls and MSCs with M2 macrophages.

Regarding BMP-2, higher concentrations of BMP-2 in M2 macrophages and MSC co-culture medium were observed after 7 days (1209.85 ± 156.74 pg/ml) and 14 days (497.88 ± 61.80 pg/ml) compared to conditioned medium from MSC monoculture (748.03 ± 66.49 pg/ml at day 7 (*P* < 0.001) and 247.52 ± 19.95 pg/ml at day 14 (*P* < 0.05), respectively (Fig. 7e). No significant differences were observed regarding BMP-2 secretion in conditioned medium between different phenotypes of macrophages, even though significantly higher BMP-2 gene expression in M2 macrophages was observed (Fig. 7f, g). BMP-2 expression in macrophage-MSC co-culture showed significantly higher values (around 4-fold) with M2 macrophages on day 7 and day 14 compared to MSC monoculture (Fig. 7h).

## Discussion

The bone healing process after injury involves interactions of multiple cell types, including osteoprogenitor cells, such as MSCs, and inflammatory cells, such as monocytes/macrophages. Although this interaction has been indicated to be critical for bone formation and related to a macrophage phenotype switch, the mechanisms involved still remain unclear. In the present study, we generated and characterized three types of macrophages and demonstrated their differential effects on the behavior of MSCs. M2 macrophages increased the mineralization of co-cultured MSCs, and this effect was proportional to the ratio of macrophages to MSCs. In contrast, M0 and M1 macrophages showed opposite effects at certain ratios. Furthermore, several potential osteogenic factors were proved to be involved in interaction between diverse macrophage subtypes and MSCs, which stimulated their osteogenic differentiation.

Macrophage phenotypes ranging from M0 to M1 and M2 were generated and characterized before co-culture. The selected cytokine secretion profiles (TNF-α, TGF-β, and IL-10), which were previously shown to discriminate M0, M1, and M2 macrophages (Freytes et al. 2013), demonstrated the successful polarization of different types of macrophages. However, unambiguous classification of macrophage phenotypes is problematic due to non-specific staining of different types of macrophages for M1 (CCR7) and M2 (CD36) markers. The quantification of relative fluorescence intensity facilitated the assignment of markers toward M1 or M2 predominance and provided an additional method to interpret macrophage subtypes. With this macrophage subtype characterization, we initiated macrophage/MSC co-cultures, during which macrophages were shown to influence MSC behavior and vice versa. As a result of these dynamics, fluctuations in macrophage subtypes can occur during co-culture, and hence maintenance of the condition at cell seeding over the course of 4 weeks cell culture is unlikely. Consequently, the pan-macrophage marker, CD68, was used to assess the distribution and viability of seeded macrophages due to the difficulty of using specific markers for different subtypes of macrophages. A larger number of macrophages were observed for M0 macrophages and MSCs in direct co-culture after 4 weeks. Additionally, different proliferation rates and osteogenic behavior of MSCs were found with three types of macrophages in direct and indirect co-cultures. These findings indicate dynamic interactions between MSCs and different types of macrophages over the co-culture period.

M2 macrophages were shown to promote osteogenic differentiation of MSCs isolated from three different donors, evidenced by the significantly higher mineralization capacity compared to MSC monoculture, both in direct and indirect co-cultures. This finding corroborates data from previous studies, which indicated that M2 macrophages stimulate the mineralization capacity of MSCs (Chen et al. 2014a, b; Fernandes et al. 2013; Gong et al. 2016; Horwood 2016). However, several pieces of contrasting data on M1 macrophage effects on MSC mineralization have been reported (Guihard et al. 2012; Loi et al. 2016). This discrepancy probably relates to experimental design differences. For instance, these previous studies did not use actual co-cultures but macrophage-conditioned medium for the culture of MSCs. Consequently, the dynamic bi-directional cellular interactions between MSCs and macrophages were not operative. Furthermore, characterization of macrophages was not reported in some studies to insure certain subtypes. Finally, the used cells for co-culture studies were dissimilar from several perspectives influencing mineralization, including MSC differentiation status (i.e. osteoprogenitors vs. mature osteoblasts), origin (i.e. adipose tissue vs. bone marrow) (Ivanova-Todorova et al. 2009) and donor characteristics (Wu et al. 2014), monocytes/macrophage characteristics (e.g., CD16 surface marker expression) (Nicolaidou et al. 2009), and co-culture cell ratios (Nicolaidou et al. 2012). This study used THP-1 monocytes activated by PMA and then polarized by LPS and IFN γ or IL4 and IL13 to reflect the M0, M1 and M2 macrophages that may occur during the bone healing in vivo. As an immortalized human cell line, THP-1 cells are characterized to retain all necessary markers and morphologic features to be qualified as a monocyte cell population (Tsuchiya et al. 1982). Under certain microenvironments, they can undergo differentiation and polarization into functional, mature macrophages. A cell line is needed here to address multivariate research questions that require large numbers of cells and also for high reproducibility of results, which was not practical and ideal for primary monocytes isolated from peripheral blood. In addition, two-dimensional co-culture was utilized in the present study to simplify the experimental complexity. A three-dimensional co-culture is ongoing to more closely mimic the real cell–cell interaction microenvironment. In the following study, an ectopic and an orthotopic in vivo model will also be conducted to assess the clinical relevance of our findings in the context of bone formation and bone repair capacity. The ratio of M2 macrophages to MSCs in the direct co-culture showed a correlation with the extent of mineralization, and an imbalance in favor of M2 macrophages even significantly increased mineralization compared to MSC monoculture controls. In our preliminary experiments (data not shown), a 10:1 ratio of M2 macrophages to MSCs showed significantly lower mineralization compared to MSC monoculture controls. Given our experimental set-up for direct co-cultures with equal numbers of MSCs for all experimental groups, this ratio-dependency suggests an optimally effective (in vitro) cytokine secretion profile. In addition, it is worthy of note that M1 macrophages with MSCs at the ratio of 1:1 slightly promoted the mineralization capacity of MSCs as well. During this co-culture process, all types of macrophages promoted the proliferation of MSCs in the first week. M0 and M2 macrophages showed this beneficial effect even after 4 weeks. On the other hand, M0 and M1 macrophages significantly promoted the osteogenic differentiation of co-cultured MSCs in the early and middle stages of osteogenesis, evidenced by high ALP activity and high gene expression of early-stage osteogenic markers such as Runx2, ALP, Col I at the early time points. In contrast, M2 macrophages showed delayed stimulatory effects on the osteogenic gene expression profile of co-cultured MSCs, with a 10-fold increased expression of BSP II on day 14. This finding corroborates the results of Omar et al. and Loi et.al, who showed that pro-inflammatory macrophages could promote the early osteogenic differentiation of bone marrow-derived MSCs and this effect was further enhanced by macrophage phenotype modulation from M1 to M2 via IL-4 treatment 72 h after seeding (Loi et al. 2016; Omar et al. 2011). Considering these findings, it could be speculated that an optimized timing of M1 and M2 macrophage appearance exists to achieve the maximum osteogenesis of co-cultured MSCs. This hypothesis was also postulated by other researchers, but needs further investigation (Horwood 2016; Schlundt et al. 2015a, b; Wu et al. 2013).

A vital role of soluble factors in the osteoinductive effects of M2 macrophages on MSCs was found because the calcium content from indirect co-culture (120.55 ± 10.09 μg/ml) was comparable to that from MSC and M2 macrophages direct co-culture (146.84 ± 12.31 μg/ml) at the same ratio. BMP-2 and OSM are most likely stimulatory molecular candidates based on previous studies (Chen et al. 2014a, b; Ekström et al. 2013; Fernandes et al. 2013; Guihard et al. 2012; Nicolaidou et al. 2012). Several studies have found that monocytes/macrophages enhanced osteogenic differentiation of MSCs in a manner dependent on an OSM signaling pathway (Fernandes et al. 2013; Guihard et al. 2012; Nicolaidou et al. 2012). In this study, M0 and M1 macrophages, but not M2 macrophages, were found to express and secrete higher OSM levels at the early time points to drive the osteogenic differentiation of co-cultured MSCs through the OSM-OSMR signaling pathway. On the other hand, higher BMP-2 secretion was observed only for MSC and M2 macrophage co-cultures, whereas BMP-2 secretion in the three types of macrophages was not significantly different. We further confirmed the increased endogenous BMP-2 secretion of MSCs when co-cultured with M2 macrophages. Two studies also proved monocytes/macrophages acting via exosomes or soluble factors on MSCs to induce autologous BMP-2 production (Ekström et al. 2013; Omar et al. 2011). This finding, however, was contradictory to the few previous reports, which demonstrated the beneficial effect of exogenous BMP-2 (Chen et al. 2014a, b; Pirraco et al. 2013). However, it needs to be emphasized that only conditioned medium was used and endogenous BMP-2 from MSCs was not tested in these studies. Nonetheless, in the present study, the possibility that BMP-2 expression in M2 macrophages was enhanced when co-cultured with MSCs cannot be excluded.

In the present study, all macrophage subtypes promoted the osteogenic differentiation of MSCs, albeit to a different extent and at different stages during co-culture. This finding challenges the traditional knowledge about macrophages and inflammation, in which macrophages were generally considered to adversely affect the bone healing process. Since at an organismal physiological level inflammatory signals resulting from bone tissue injury or surgery, and the implanted biomaterial mediate the differentiation of monocytes into different types of macrophages, our findings provide new impetus for the future design of supporting scaffolds and cell selection for treatment of bone defects. For instance, hydrophilic nanostructured surfaces have been shown to drive M2 macrophage polarization and improve osseointegration (Ma et al. 2014a), and enhancement of M2 phenotype in bone defects further improved bone healing (Schlundt et al. 2015a, b). Furthermore, MSCs, irrespective of their origin, have been indicated to function as immunomodulators to macrophages beyond their differentiation potential in tissue regeneration (Nauta and Fibbe 2007; Swartzlander et al. 2015). Their effect on bone regeneration through immunoregulation mechanisms is worth investigating in the future.

## Conclusion

This work systematically studied the effects of different macrophage subtypes on the osteogenic differentiation of adipose tissue MSCs. We found that M2 macrophages had a beneficial effect on ADMSCs mineralization by promoting their proliferation and osteogenic differentiation. In contrast, this enhanced mineralization effect was not observed for ADMSCS co-cultured with M0 and M1 macrophages at certain ratios, although both of them were able to promote the early osteogenic process. Furthermore, indirect co-cultures demonstrated that the stimulatory effect was mediated by soluble factors, in which autocrine BMP-2 and OSM osteogenic factors were involved. Our findings not only elucidate the critical role of macrophages in the osteogenic differentiation process of osteoprogenitor cells but also provide important considerations for the implementation of macrophage–osteoprogenitor cell interactions in the development of bone regenerative treatments.

## Electronic supplementary material

Below is the link to the electronic supplementary material.

Fig. S1 Cell proliferation and ALP activity of MSCs from donor 1 and 2 indirectly co-cultured with M0, M1 and M2 macrophages. MSCs were monocultured and indirectly co-cultured with three types of macrophages and their proliferation was determined by DNA content assay (**a**, **c**) and their osteogenic differentiation was determined by ALP activity assay (**b**, **d**). Statistical analysis was performed by one-way ANOVA with Dunnett’s post-test. *n* = 4, *P ≤ 0.05, **P ≤ 0.01, ***P ≤ 0.001. (GIF 51 kb)

High resolution image (TIF 4982 kb)

Table S1 (DOCX 13 kb)
