# Supplementary figures and images for: Macrophage type modulates osteogenic differentiation of adipose tissue MSCs
Source: Cell Tissue Res. 2017 Mar 30;369(2):273–86. doi: 10.1007/s00441-017-2598-8 (PMC5552848; doi:10.1007/s00441-017-2598-8)

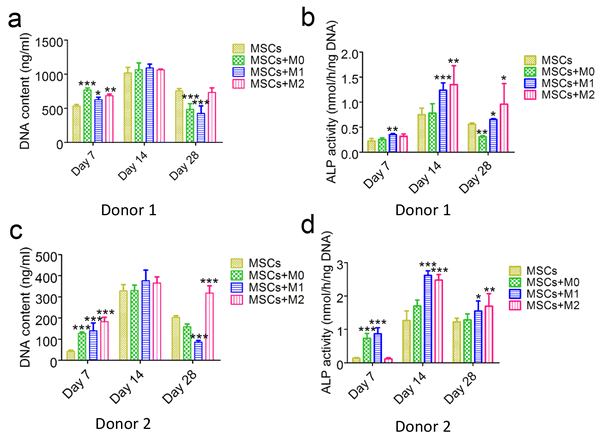

Supplement: Supplementary file 1 — Cell proliferation and ALP activity of MSCs from donor 1 and 2 indirectly co-cultured with M0, M1 and M2 macrophages. MSCs were monocultured and indirectly co-cultured with three types of macrophages and their proliferation was determined by DNA content assay (a, c) and their osteogenic differentiation was determined by ALP activity assay (b, d). Statistical analysis was performed by one-way ANOVA with Dunnett’s post-test. n = 4, *P ≤ 0.05, **P ≤ 0.01, ***P ≤ 0.001. (GIF 51 kb) [file 441_2017_2598_Fig8_ESM.gif]

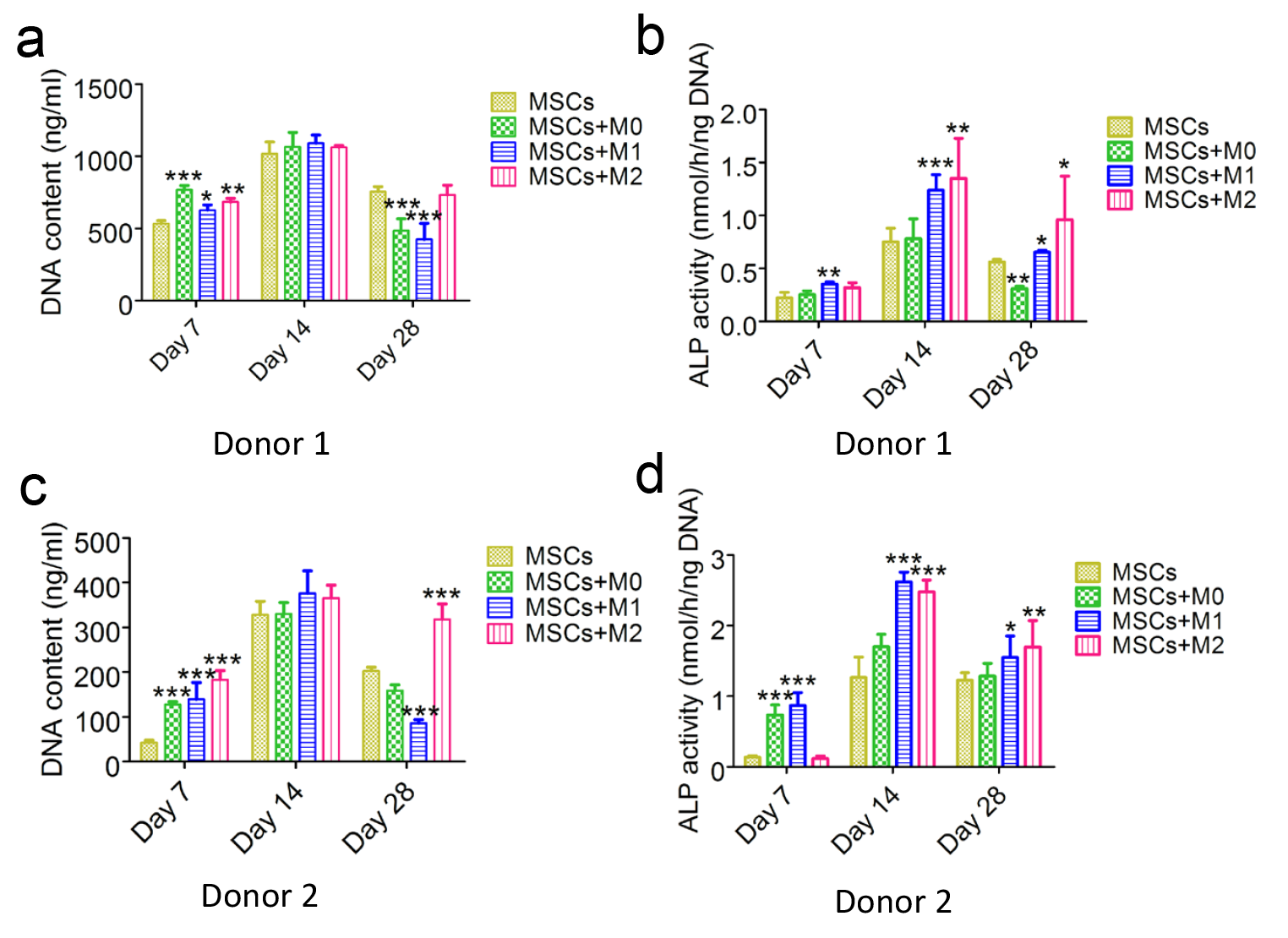

Supplement: Supplementary file 2 — (TIF 4982 kb) [file 441_2017_2598_MOESM1_ESM.tif]
