# Supplementary material for: Macrophage type modulates osteogenic differentiation of adipose tissue MSCs
Source: Cell Tissue Res. 2017 Mar 30;369(2):273–86. doi: 10.1007/s00441-017-2598-8 (PMC5552848; doi:10.1007/s00441-017-2598-8)
Supplement: Supplementary file 3 — (DOCX 13 kb) [file 441_2017_2598_MOESM2_ESM.docx]

**Tab. S1 Primers used for RT-PCR**

| Gene Name | Forward Primer | Reverse Primer |
| --- | --- | --- |
| GAPDH | CTCTGCTCCTCCTGTTCGACA | ACGACCAAATCCGTTGACTC |
| RUNX2 | GGAGTGGACGAGGCAAGAGTTT | AGCTTCTGTCTGTGCCTTCTGG |
| COL I | GGT GTAAGCGGTGGTGGTTAT | GCTGGGATGTTTTCAGGTTGG |
| ALP | CCCAAAGGCTTCTTCTTG | CTGGTAGTTGTTGTG AGCAT |
| OCN | GACTGTGACGAGTTGGCTGA | CTGGAGAGGAGCAGAACTGG |
| BSP | AACCTACAACCCCACCACAA | AGGTTCCCCGTTCTCACTTT |
| OSM | TACCATCGCTTCATGCACTC | GGAGGAGGTAGAGGGGTCTG |
| OSMR | TGGCTCTATTTGCAGTCTTTCA | ATGCCTTCACTGACATTTCCTT |
| BMP-2 | ATGAAGAATCTTTGGAAGAACTAC | GGTGATGGAAACTGCTATTG |
